# Frequent Mutations in EGFR, KRAS and TP53 Genes in Human Lung Cancer Tumors Detected by Ion Torrent DNA Sequencing

**DOI:** 10.1371/journal.pone.0095228

**Published:** 2014-04-23

**Authors:** Xin Cai, Jianhui Sheng, Chuanning Tang, Vijayalakshmi Nandakumar, Hua Ye, Hong Ji, Haiying Tang, Yu Qin, Hongwei Guan, Feng Lou, Dandan Zhang, Hong Sun, Haichao Dong, Guangchun Zhang, Zhiyuan Liu, Zhishou Dong, Baishuai Guo, He Yan, Chaowei Yan, Lu Wang, Ziyi Su, Yangyang Li, Lindsey Jones, Xue F. Huang, Si-Yi Chen, Taihua Wu, Hongli Lin

**Affiliations:** 1 The First Affiliated Hospital of Dalian Medical University, Dalian, Liaoning, China; 2 San Valley Biotechnology Incorporated, Beijing, China; 3 Norris Comprehensive Cancer Center, Department of Molecular Microbiology and Immunology, Keck School of Medicine, University of Southern California Los Angeles, Los Angeles, California, United States of America; The University of Hong Kong, China

## Abstract

Lung cancer is the most common malignancy and the leading cause of cancer deaths worldwide. While smoking is by far the leading cause of lung cancer, other environmental and genetic factors influence the development and progression of the cancer. Since unique mutations patterns have been observed in individual cancer samples, identification and characterization of the distinctive lung cancer molecular profile is essential for developing more effective, tailored therapies. Until recently, personalized DNA sequencing to identify genetic mutations in cancer was impractical and expensive. The recent technological advancements in next-generation DNA sequencing, such as the semiconductor-based Ion Torrent sequencing platform, has made DNA sequencing cost and time effective with more reliable results. Using the Ion Torrent Ampliseq Cancer Panel, we sequenced 737 loci from 45 cancer-related genes to identify genetic mutations in 76 human lung cancer samples. The sequencing analysis revealed missense mutations in KRAS, EGFR, and TP53 genes in the breast cancer samples of various histologic types. Thus, this study demonstrates the necessity of sequencing individual human cancers in order to develop personalized drugs or combination therapies to effectively target individual, breast cancer-specific mutations.

## Introduction

Lung cancer is the most common malignancy worldwide, and also the leading cause of cancer related deaths. In 2008, an estimated 1.61 million new cases were reported globally, accounting for 12.7% of all new cancers [1]. Additionally, roughly 1.38 million deaths (18.2% of total cancer deaths) were reported around the world [2]. In China, lung cancer has the highest incidence of all new cancer cases in both men and women (21.7% in 2008) with more than a 24.9% mortality rate [2]. Women in China reported only a slightly higher incidence of lung cancer over breast cancer this same year; however, the mortality rate of lung cancer is more than 3 times higher than that of breast cancer (20.2% vs. 6.1%, respectively) [2]. Lung cancer often exhibits non-specific symptoms, and diagnosis often occurs at an advanced stage or after metastasis has already occurred [3]. While efforts continue to improve early diagnosis and treatment of lung cancer, the staggering incidence, poor prognosis, and considerable mortality rate prevails.

The leading cause of lung cancer is cigarette smoking, and increased exposure is directly correlated with an increased risk of developing lung cancer [4]. 85–90% of lung cancer deaths are associated with smoking, and current smokers are 15 times more likely to die from lung cancer than never-smokers [5]. There are two major forms of lung cancer: non-small-cell lung cancer (NSCLC) and small-cell lung cancer (SCLC). NSCLC, which accounts for roughly 85% of all lung cancers, can be further divided into three major histologic subtypes: squamous-cell carcinoma (SCC), adenocarcinoma, and large-cell lung cancer. While smoking can be attributed to all forms of lung cancer, it is most commonly linked to SCLC and SCC. Never-smokers, on the other hand, are most commonly diagnosed with adenocarcinoma [3], [5]. Interestingly, only 10–24% of smokers develop lung cancer, indicating the importance of other environmental and individual genetic factors [6], [7]. Aside from tobacco smoke, other etiologic agents and risk factors have been identified, including occupation, exposure to second-hand smoke, asbestos, radon gas, and air pollution, in addition to genetic factors [8]–[10]. Roughly 10–15% of lung cancers arise in patients that report never having smoked and these cancers do so spontaneously with an accumulation of genetic and epigenetic changes [5].

Despite ongoing efforts to improve screening and treatment of lung cancers, the prognosis of patients with most forms of lung cancer remains poor [3]. Because the genetic and environmental factors causing lung cancer vary widely, each tumor has the potential to exhibit a unique gene mutation profile. As such, accumulating evidence suggests that individualized, tailored therapies are essential for effective treatment against lung cancers. This can be accomplished by profiling an individual's cancer genome in order to dissect the oncogenic mechanisms that regulate the progression of the cancer. Recently, a new technology based on semiconductor sequencing called Ion Torrent sequencing [11] is tackling many of the issues associated with other sequencing methods, namely the cost, time, and overall practicality of individualized genome sequencing. In this study, we have used Ion Torrent sequencing to analyze 76 clinical lung cancer samples to identify the genetic mutations in 737 loci of 45 known cancer-related genes.

## Results

### Mutation analysis of human lung cancer tumors with Ion Ampliseq Cancer Panel

A total of 76 Lung cancer samples (**Table 1**) was analyzed using Ion Torrent Ampliseq Cancer Panel to identify mutations in 737 loci of 45 oncogenes and tumor suppressor genes in human lung cancers. These Lung cancer samples were all from Chinese patients ranging from 28–80 years old represented by 40 men with a mean age of 62 years and 36 women with a mean age of 59 years.

**Table 1:** Detected mutations (including Missense point mutations/deletion/insertion) in 45 genes (737 loci) of 76 human lung cancer samples.

Genes	Number of samples with mutations in 76 samples (Mutation frequency)	Number of female samples with mutations (Mutation frequency in 36 female samples)	Number of male samples with mutations (Mutation frequency in 40 male samples)
ABL1	0(0.0%)	0(0.0%)	0(0.0%)
AKT1	0(0.0%)	0(0.0%)	0(0.0%)
ALK	0(0.0%)	0(0.0%)	0(0.0%)
APC	0(0.0%)	0(0.0%)	0(0.0%)
ATM	0(0.0%)	0(0.0%)	0(0.0%)
BRAF	2(2.6%)	1(2.8%)	1(2.5%)
CDH1	0(0.0%)	0(0.0%)	0(0.0%)
CDKN2A	0(0.0%)	0(0.0%)	0(0.0%)
CSF1R	0(0.0%)	0(0.0%)	0(0.0%)
CTNNB1	3(3.9%)	3(8.3%)	0(0.0%)
EGFR	32(42.1%)	22(61.1%)	10(25.0%)
ERBB2	1(1.3%)	0(0.0%)	1(2.5%)
ERBB4	0(0.0%)	0(0.0%)	0(0.0%)
FBXW7	0(0.0%)	0(0.0%)	0(0.0%)
FGFR1	0(0.0%)	0(0.0%)	0(0.0%)
FGFR2	0(0.0%)	0(0.0%)	0(0.0%)
FGFR3	0(0.0%)	0(0.0%)	0(0.0%)
FLT3	0(0.0%)	0(0.0%)	0(0.0%)
GNAS	0(0.0%)	0(0.0%)	0(0.0%)
HNF1A	0(0.0%)	0(0.0%)	0(0.0%)
HRAS	0(0.0%)	0(0.0%)	0(0.0%)
IDH1	0(0.0%)	0(0.0%)	0(0.0%)
JAK3	0(0.0%)	0(0.0%)	0(0.0%)
KDR	0(0.0%)	0(0.0%)	0(0.0%)
KIT	0(0.0%)	0(0.0%)	0(0.0%)
KRAS	4(5.3%)	1(2.8%)	3(7.5%)
MET	0(0.0%)	0(0.0%)	0(0.0%)
MLH1	0(0.0%)	0(0.0%)	0(0.0%)
MPL	0(0.0%)	0(0.0%)	0(0.0%)
NOTCH1	0(0.0%)	0(0.0%)	0(0.0%)
NPM1	0(0.0%)	0(0.0%)	0(0.0%)
NRAS	0(0.0%)	0(0.0%)	0(0.0%)
PDGFRA	0(0.0%)	0(0.0%)	0(0.0%)
PIK3CA	2(2.6%)	0(0.0%)	2(5.0%)
PTEN	1(1.3%)	1(2.8%)	0(0.0%)
PTPN11	0(0.0%)	0(0.0%)	0(0.0%)
RB1	0(0.0%)	0(0.0%)	0(0.0%)
RET	0(0.0%)	0(0.0%)	0(0.0%)
SMAD4	1(1.3%)	1(2.8%)	0(0.0%)
SMARCB1	0(0.0%)	0(0.0%)	0(0.0%)
SMO	0(0.0%)	0(0.0%)	0(0.0%)
SRC	0(0.0%)	0(0.0%)	0(0.0%)
STK11	0(0.0%)	0(0.0%)	0(0.0%)
TP53	17(22.4%)	6(16.7%)	11(27.5%)
VHL	0(0.0%)	0(0.0%)	0(0.0%)

**Figure 1:**
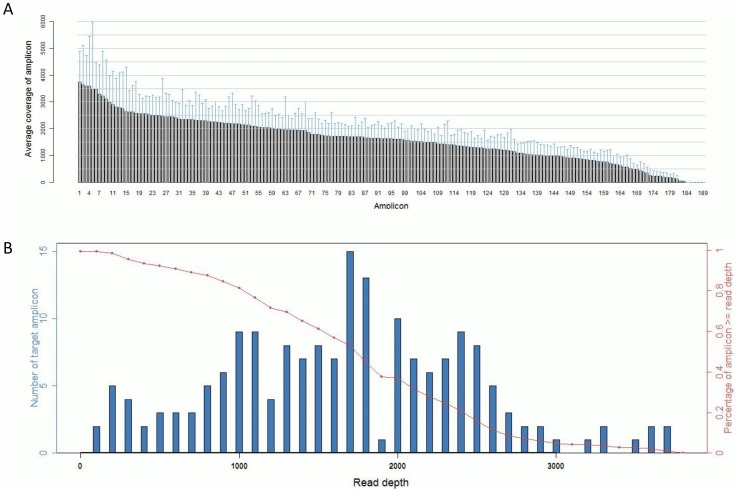
Sequence read distribution across 189 amplicons generated from 76 FFPE specimens, normalized to 300,000 reads per sample. A. Distribution of average coverage of each amplicon. Data are showed as mean ±SD. B. Number of amplicons with a given read depth, sorted in bins of 100 reads. (blue bars present number of target amplicons within read depth, red line presents % of target amplicons > =  read depth).

The sequenced data were processed and mutations identified using Ion Torrent Suite Software v3.0 with a plug-in “variant caller”. In order to eliminate error base calling, three filtering steps were used to generate reliable variant calling as described in the Materials and Methods. The Sequence read distribution across 189 amplicons generated from 76 FFPE specimens were normalized to 300,000 reads per sample (**Fig. 1**). Using a strict standard variant calling, we identified mutations in the following genes as listed in **Table 1**: BRAF, EGFR, ERBB2, KRAS, PIK3CA, PTEN, SMAD4, and TP53.

The samples were classified based on their origin as lung adenocarcinoma, lung large cell carcinoma, lung squamous cell carcinoma and lung neuroendocrine carcinoma. The different stages the cancers have progressed to were scored based on ‘American Joint Committee on Cancer/**T**umor size, Lymph **N**odes affected, **M**etastases (AJCC/TNM) ' system (Ia, Ib, IIa, IIb, IIIa, IIIb) and as metastasizing and non-metastasizing lung cancers. Also, cancers were sorted out as from heavy smokers, light-smokers and non-smokers to check the correlation of smoking with the accumulation of these mutations. The detailed list of missense point mutations, insertions, and deletions profiled on 737 loci of 76 lung cancer samples is provided in **Table S1**.

Out of the mutations identified in our sample set, BRAF (2.6%), EGFR (42.1%), ERBB2 (1.3%), KRAS (5.3%), PIK3CA (2.6%), PTEN (1.3%), SMAD4 (1.3%), and TP53 (22.4%) incurred the highest rates of mutations (**Table 2**). The mutation frequencies at their different differentiation levels (**Table 3**), at different AJCC staging (**Table 4**), of the metastatic and non-metastatic lung cancers (**Table 5**) and from patients with different smoking habits (**Table 6**) are outlined in the Tables. Detailed sequencing analysis in the exons and functional domains of these genes was hence performed.

**Table 2:** Mutations (including Missense point mutations/deletion/insertion) frequencies in 45 genes (737 loci) in lung adenocarcinoma (AC), lung large cell carcinoma, lung squamous cell carcinoma and other two lung cancer samples (lung neuroendocrine carcinoma and unknown lung cancer).

Genes	Number of samples with mutations in 76 samples (Mutation frequency)	Number of AC samples with mutations (Mutation frequency in 52 AC samples)	Number of SC samples with mutations (Mutation frequency in 20 SC samples)	Number of LCC samples with mutations (Mutation frequency in 2 LCC samples)	Number of other samples with mutations (Mutation frequency in 2 other samples)
ABL1	0(0.0%)	0(0.0%)	0(0.0%)	0(0.0%)	0(0.0%)
AKT1	0(0.0%)	0(0.0%)	0(0.0%)	0(0.0%)	0(0.0%)
ALK	0(0.0%)	0(0.0%)	0(0.0%)	0(0.0%)	0(0.0%)
APC	0(0.0%)	0(0.0%)	0(0.0%)	0(0.0%)	0(0.0%)
ATM	0(0.0%)	0(0.0%)	0(0.0%)	0(0.0%)	0(0.0%)
BRAF	2(2.6%)	1(1.9%)	1(5.0%)	0(0.0%)	0(0.0%)
CDH1	0(0.0%)	0(0.0%)	0(0.0%)	0(0.0%)	0(0.0%)
CDKN2A	0(0.0%)	0(0.0%)	0(0.0%)	0(0.0%)	0(0.0%)
CSF1R	0(0.0%)	0(0.0%)	0(0.0%)	0(0.0%)	0(0.0%)
CTNNB1	3(3.9%)	3(5.8%)	0(0.0%)	0(0.0%)	0(0.0%)
EGFR	32(42.1%)	30(57.7%)	1(5.0%)	0(0.0%)	1(50.0%)
ERBB2	1(1.3%)	1(1.9%)	0(0.0%)	0(0.0%)	0(0.0%)
ERBB4	0(0.0%)	0(0.0%)	0(0.0%)	0(0.0%)	0(0.0%)
FBXW7	0(0.0%)	0(0.0%)	0(0.0%)	0(0.0%)	0(0.0%)
FGFR1	0(0.0%)	0(0.0%)	0(0.0%)	0(0.0%)	0(0.0%)
FGFR2	0(0.0%)	0(0.0%)	0(0.0%)	0(0.0%)	0(0.0%)
FGFR3	0(0.0%)	0(0.0%)	0(0.0%)	0(0.0%)	0(0.0%)
FLT3	0(0.0%)	0(0.0%)	0(0.0%)	0(0.0%)	0(0.0%)
GNAS	0(0.0%)	0(0.0%)	0(0.0%)	0(0.0%)	0(0.0%)
HNF1A	0(0.0%)	0(0.0%)	0(0.0%)	0(0.0%)	0(0.0%)
HRAS	0(0.0%)	0(0.0%)	0(0.0%)	0(0.0%)	0(0.0%)
IDH1	0(0.0%)	0(0.0%)	0(0.0%)	0(0.0%)	0(0.0%)
JAK3	0(0.0%)	0(0.0%)	0(0.0%)	0(0.0%)	0(0.0%)
KDR	0(0.0%)	0(0.0%)	0(0.0%)	0(0.0%)	0(0.0%)
KIT	0(0.0%)	0(0.0%)	0(0.0%)	0(0.0%)	0(0.0%)
KRAS	4(5.3%)	4(7.7%)	0(0.0%)	0(0.0%)	0(0.0%)
MET	0(0.0%)	0(0.0%)	0(0.0%)	0(0.0%)	0(0.0%)
MLH1	0(0.0%)	0(0.0%)	0(0.0%)	0(0.0%)	0(0.0%)
MPL	0(0.0%)	0(0.0%)	0(0.0%)	0(0.0%)	0(0.0%)
NOTCH1	0(0.0%)	0(0.0%)	0(0.0%)	0(0.0%)	0(0.0%)
NPM1	0(0.0%)	0(0.0%)	0(0.0%)	0(0.0%)	0(0.0%)
NRAS	0(0.0%)	0(0.0%)	0(0.0%)	0(0.0%)	0(0.0%)
PDGFRA	0(0.0%)	0(0.0%)	0(0.0%)	0(0.0%)	0(0.0%)
PIK3CA	2(2.6%)	1(1.9%)	1(5.0%)	0(0.0%)	0(0.0%)
PTEN	1(1.3%)	1(1.9%)	0(0.0%)	0(0.0%)	0(0.0%)
PTPN11	0(0.0%)	0(0.0%)	0(0.0%)	0(0.0%)	0(0.0%)
RB1	0(0.0%)	0(0.0%)	0(0.0%)	0(0.0%)	0(0.0%)
RET	0(0.0%)	0(0.0%)	0(0.0%)	0(0.0%)	0(0.0%)
SMAD4	1(1.3%)	1(1.9%)	0(0.0%)	0(0.0%)	0(0.0%)
SMARCB1	0(0.0%)	0(0.0%)	0(0.0%)	0(0.0%)	0(0.0%)
SMO	0(0.0%)	0(0.0%)	0(0.0%)	0(0.0%)	0(0.0%)
SRC	0(0.0%)	0(0.0%)	0(0.0%)	0(0.0%)	0(0.0%)
STK11	0(0.0%)	0(0.0%)	0(0.0%)	0(0.0%)	0(0.0%)
TP53	17(22.4%)	10(19.2%)	6(30.0%)	1(50.0%)	0(0.0%)
VHL	0(0.0%)	0(0.0%)	0(0.0%)	0(0.0%)	0(0.0%)

**Table 3:** Mutations (including Missense point mutations/deletion/insertion) frequencies in 45 genes (737 loci) of human lung cancer patients at different differentiation levels.

Genes	Number of samples with mutations (Mutation frequency in 76 samples)	Number of high differentiation samples with mutations (Mutation frequency in 15 samples)	Number of low differentiation samples with mutations (Mutation frequency in 33 samples)	Number of middle differentiation samples with mutations (Mutation frequency in 27 samples)	Number of unknown samples with mutations (Mutation frequency in 1 samples)
ABL1	0(0.0%)	0(0.0%)	0(0.0%)	0(0.0%)	0(0.0%)
AKT1	0(0.0%)	0(0.0%)	0(0.0%)	0(0.0%)	0(0.0%)
ALK	0(0.0%)	0(0.0%)	0(0.0%)	0(0.0%)	0(0.0%)
APC	0(0.0%)	0(0.0%)	0(0.0%)	0(0.0%)	0(0.0%)
ATM	0(0.0%)	0(0.0%)	0(0.0%)	0(0.0%)	0(0.0%)
BRAF	2(2.6%)	0(0.0%)	1(3.0%)	1(3.7%)	0(0.0%)
CDH1	0(0.0%)	0(0.0%)	0(0.0%)	0(0.0%)	0(0.0%)
CDKN2A	0(0.0%)	0(0.0%)	0(0.0%)	0(0.0%)	0(0.0%)
CSF1R	0(0.0%)	0(0.0%)	0(0.0%)	0(0.0%)	0(0.0%)
CTNNB1	3(3.9%)	0(0.0%)	0(0.0%)	3(11.1%)	0(0.0%)
EGFR	32(42.1%)	13(86.7%)	5(15.2%)	14(51.9%)	0(0.0%)
ERBB2	1(1.3%)	0(0.0%)	1(3.0%)	0(0.0%)	0(0.0%)
ERBB4	0(0.0%)	0(0.0%)	0(0.0%)	0(0.0%)	0(0.0%)
FBXW7	0(0.0%)	0(0.0%)	0(0.0%)	0(0.0%)	0(0.0%)
FGFR1	0(0.0%)	0(0.0%)	0(0.0%)	0(0.0%)	0(0.0%)
FGFR2	0(0.0%)	0(0.0%)	0(0.0%)	0(0.0%)	0(0.0%)
FGFR3	0(0.0%)	0(0.0%)	0(0.0%)	0(0.0%)	0(0.0%)
FLT3	0(0.0%)	0(0.0%)	0(0.0%)	0(0.0%)	0(0.0%)
GNAS	0(0.0%)	0(0.0%)	0(0.0%)	0(0.0%)	0(0.0%)
HNF1A	0(0.0%)	0(0.0%)	0(0.0%)	0(0.0%)	0(0.0%)
HRAS	0(0.0%)	0(0.0%)	0(0.0%)	0(0.0%)	0(0.0%)
IDH1	0(0.0%)	0(0.0%)	0(0.0%)	0(0.0%)	0(0.0%)
JAK3	0(0.0%)	0(0.0%)	0(0.0%)	0(0.0%)	0(0.0%)
KDR	0(0.0%)	0(0.0%)	0(0.0%)	0(0.0%)	0(0.0%)
KIT	0(0.0%)	0(0.0%)	0(0.0%)	0(0.0%)	0(0.0%)
KRAS	4(5.3%)	0(0.0%)	2(6.1%)	2(7.4%)	0(0.0%)
MET	0(0.0%)	0(0.0%)	0(0.0%)	0(0.0%)	0(0.0%)
MLH1	0(0.0%)	0(0.0%)	0(0.0%)	0(0.0%)	0(0.0%)
MPL	0(0.0%)	0(0.0%)	0(0.0%)	0(0.0%)	0(0.0%)
NOTCH1	0(0.0%)	0(0.0%)	0(0.0%)	0(0.0%)	0(0.0%)
NPM1	0(0.0%)	0(0.0%)	0(0.0%)	0(0.0%)	0(0.0%)
NRAS	0(0.0%)	0(0.0%)	0(0.0%)	0(0.0%)	0(0.0%)
PDGFRA	0(0.0%)	0(0.0%)	0(0.0%)	0(0.0%)	0(0.0%)
PIK3CA	2(2.6%)	0(0.0%)	1(3.0%)	1(3.7%)	0(0.0%)
PTEN	1(1.3%)	0(0.0%)	0(0.0%)	1(3.7%)	0(0.0%)
PTPN11	0(0.0%)	0(0.0%)	0(0.0%)	0(0.0%)	0(0.0%)
RB1	0(0.0%)	0(0.0%)	0(0.0%)	0(0.0%)	0(0.0%)
RET	0(0.0%)	0(0.0%)	0(0.0%)	0(0.0%)	0(0.0%)
SMAD4	1(1.3%)	0(0.0%)	0(0.0%)	1(3.7%)	0(0.0%)
SMARCB1	0(0.0%)	0(0.0%)	0(0.0%)	0(0.0%)	0(0.0%)
SMO	0(0.0%)	0(0.0%)	0(0.0%)	0(0.0%)	0(0.0%)
SRC	0(0.0%)	0(0.0%)	0(0.0%)	0(0.0%)	0(0.0%)
STK11	0(0.0%)	0(0.0%)	0(0.0%)	0(0.0%)	0(0.0%)
TP53	17(22.4%)	3(20.0%)	7(21.2%)	7(25.9%)	0(0.0%)
VHL	0(0.0%)	0(0.0%)	0(0.0%)	0(0.0%)	0(0.0%)

**Table 4:** Mutations (including Missense point mutations/deletion/insertion) frequencies in 45 genes (737 loci) of lung cancer patients at different stagings according to AJCC staging.

Genes	Number of samples with mutations (Mutation frequency in 76 samples)	Number of stage 1B samples with mutations (Mutation frequency in 18 samples)	Number of stage 2A samples with mutations (Mutation frequency in 3 samples)	Number of stage 2B samples with mutations (Mutation frequency in 9 samples)	Number of stage 3A samples with mutations (Mutation frequency in 26 samples)	Number of stage 3B samples with mutations (Mutation frequency in 8 samples)	Number of stage 4 samples with mutations (Mutation frequency in 12 samples)
ABL1	0(0.0%)	0(0.0%)	0(0.0%)	0(0.0%)	0(0.0%)	0(0.0%)	0(0.0%)
AKT1	0(0.0%)	0(0.0%)	0(0.0%)	0(0.0%)	0(0.0%)	0(0.0%)	0(0.0%)
ALK	0(0.0%)	0(0.0%)	0(0.0%)	0(0.0%)	0(0.0%)	0(0.0%)	0(0.0%)
APC	0(0.0%)	0(0.0%)	0(0.0%)	0(0.0%)	0(0.0%)	0(0.0%)	0(0.0%)
ATM	0(0.0%)	0(0.0%)	0(0.0%)	0(0.0%)	0(0.0%)	0(0.0%)	0(0.0%)
BRAF	2(2.6%)	0(0.0%)	0(0.0%)	0(0.0%)	1(3.8%)	0(0.0%)	1(8.3%)
CDH1	0(0.0%)	0(0.0%)	0(0.0%)	0(0.0%)	0(0.0%)	0(0.0%)	0(0.0%)
CDKN2A	0(0.0%)	0(0.0%)	0(0.0%)	0(0.0%)	0(0.0%)	0(0.0%)	0(0.0%)
CSF1R	0(0.0%)	0(0.0%)	0(0.0%)	0(0.0%)	0(0.0%)	0(0.0%)	0(0.0%)
CTNNB1	3(3.9%)	0(0.0%)	0(0.0%)	0(0.0%)	2(7.7%)	0(0.0%)	1(8.3%)
EGFR	32(42.1%)	10(55.6%)	1(33.3%)	5(55.6%)	9(34.6%)	3(37.5%)	4(33.3%)
ERBB2	1(1.3%)	0(0.0%)	0(0.0%)	0(0.0%)	1(3.8%)	0(0.0%)	0(0.0%)
ERBB4	0(0.0%)	0(0.0%)	0(0.0%)	0(0.0%)	0(0.0%)	0(0.0%)	0(0.0%)
FBXW7	0(0.0%)	0(0.0%)	0(0.0%)	0(0.0%)	0(0.0%)	0(0.0%)	0(0.0%)
FGFR1	0(0.0%)	0(0.0%)	0(0.0%)	0(0.0%)	0(0.0%)	0(0.0%)	0(0.0%)
FGFR2	0(0.0%)	0(0.0%)	0(0.0%)	0(0.0%)	0(0.0%)	0(0.0%)	0(0.0%)
FGFR3	0(0.0%)	0(0.0%)	0(0.0%)	0(0.0%)	0(0.0%)	0(0.0%)	0(0.0%)
FLT3	0(0.0%)	0(0.0%)	0(0.0%)	0(0.0%)	0(0.0%)	0(0.0%)	0(0.0%)
GNAS	0(0.0%)	0(0.0%)	0(0.0%)	0(0.0%)	0(0.0%)	0(0.0%)	0(0.0%)
HNF1A	0(0.0%)	0(0.0%)	0(0.0%)	0(0.0%)	0(0.0%)	0(0.0%)	0(0.0%)
HRAS	0(0.0%)	0(0.0%)	0(0.0%)	0(0.0%)	0(0.0%)	0(0.0%)	0(0.0%)
IDH1	0(0.0%)	0(0.0%)	0(0.0%)	0(0.0%)	0(0.0%)	0(0.0%)	0(0.0%)
JAK3	0(0.0%)	0(0.0%)	0(0.0%)	0(0.0%)	0(0.0%)	0(0.0%)	0(0.0%)
KDR	0(0.0%)	0(0.0%)	0(0.0%)	0(0.0%)	0(0.0%)	0(0.0%)	0(0.0%)
KIT	0(0.0%)	0(0.0%)	0(0.0%)	0(0.0%)	0(0.0%)	0(0.0%)	0(0.0%)
KRAS	4(5.3%)	1(5.6%)	0(0.0%)	1(11.1%)	0(0.0%)	1(12.5%)	1(8.3%)
MET	0(0.0%)	0(0.0%)	0(0.0%)	0(0.0%)	0(0.0%)	0(0.0%)	0(0.0%)
MLH1	0(0.0%)	0(0.0%)	0(0.0%)	0(0.0%)	0(0.0%)	0(0.0%)	0(0.0%)
MPL	0(0.0%)	0(0.0%)	0(0.0%)	0(0.0%)	0(0.0%)	0(0.0%)	0(0.0%)
NOTCH1	0(0.0%)	0(0.0%)	0(0.0%)	0(0.0%)	0(0.0%)	0(0.0%)	0(0.0%)
NPM1	0(0.0%)	0(0.0%)	0(0.0%)	0(0.0%)	0(0.0%)	0(0.0%)	0(0.0%)
NRAS	0(0.0%)	0(0.0%)	0(0.0%)	0(0.0%)	0(0.0%)	0(0.0%)	0(0.0%)
PDGFRA	0(0.0%)	0(0.0%)	0(0.0%)	0(0.0%)	0(0.0%)	0(0.0%)	0(0.0%)
PIK3CA	2(2.6%)	0(0.0%)	0(0.0%)	1(11.1%)	1(3.8%)	0(0.0%)	0(0.0%)
PTEN	1(1.3%)	0(0.0%)	0(0.0%)	1(11.1%)	0(0.0%)	0(0.0%)	0(0.0%)
PTPN11	0(0.0%)	0(0.0%)	0(0.0%)	0(0.0%)	0(0.0%)	0(0.0%)	0(0.0%)
RB1	0(0.0%)	0(0.0%)	0(0.0%)	0(0.0%)	0(0.0%)	0(0.0%)	0(0.0%)
RET	0(0.0%)	0(0.0%)	0(0.0%)	0(0.0%)	0(0.0%)	0(0.0%)	0(0.0%)
SMAD4	1(1.3%)	0(0.0%)	0(0.0%)	1(11.1%)	0(0.0%)	0(0.0%)	0(0.0%)
SMARCB1	0(0.0%)	0(0.0%)	0(0.0%)	0(0.0%)	0(0.0%)	0(0.0%)	0(0.0%)
SMO	0(0.0%)	0(0.0%)	0(0.0%)	0(0.0%)	0(0.0%)	0(0.0%)	0(0.0%)
SRC	0(0.0%)	0(0.0%)	0(0.0%)	0(0.0%)	0(0.0%)	0(0.0%)	0(0.0%)
STK11	0(0.0%)	0(0.0%)	0(0.0%)	0(0.0%)	0(0.0%)	0(0.0%)	0(0.0%)
TP53	17(22.4%)	3(16.7%)	1(33.3%)	1(11.1%)	9(34.6%)	1(12.5%)	2(16.7%)
VHL	0(0.0%)	0(0.0%)	0(0.0%)	0(0.0%)	0(0.0%)	0(0.0%)	0(0.0%)

**Table 5:** Mutations (including Missense point mutations/deletion/insertion) frequencies in 45 genes (737 loci) in metastasis and non-metastasis lung cancer patients.

Genes	Number of samples with mutations (Mutation frequency in 76 samples)	Number of non-metastasis samples with mutations (Mutation frequency in 16 samples)	Number of local metastasis samples with mutations (Mutation frequency in 8 samples)	Number of lymph metastasis samples with mutations (Mutation frequency in 11 samples)	Number of far metastasis samples with mutations (Mutation frequency in 39 samples)	Number of unknown samples with mutations (Mutation frequency in 2 samples)
ABL1	0(0.0%)	0(0.0%)	0(0.0%)	0(0.0%)	0(0.0%)	0(0.0%)
AKT1	0(0.0%)	0(0.0%)	0(0.0%)	0(0.0%)	0(0.0%)	0(0.0%)
ALK	0(0.0%)	0(0.0%)	0(0.0%)	0(0.0%)	0(0.0%)	0(0.0%)
APC	0(0.0%)	0(0.0%)	0(0.0%)	0(0.0%)	0(0.0%)	0(0.0%)
ATM	0(0.0%)	0(0.0%)	0(0.0%)	0(0.0%)	0(0.0%)	0(0.0%)
BRAF	2(2.6%)	0(0.0%)	0(0.0%)	1(9.1%)	1(2.6%)	0(0.0%)
CDH1	0(0.0%)	0(0.0%)	0(0.0%)	0(0.0%)	0(0.0%)	0(0.0%)
CDKN2A	0(0.0%)	0(0.0%)	0(0.0%)	0(0.0%)	0(0.0%)	0(0.0%)
CSF1R	0(0.0%)	0(0.0%)	0(0.0%)	0(0.0%)	0(0.0%)	0(0.0%)
CTNNB1	3(3.9%)	0(0.0%)	1(12.5%)	0(0.0%)	2(5.1%)	0(0.0%)
EGFR	32(42.1%)	7(43.8%)	4(50.0%)	3(27.3%)	18(46.2%)	0(0.0%)
ERBB2	1(1.3%)	0(0.0%)	0(0.0%)	0(0.0%)	1(2.6%)	0(0.0%)
ERBB4	0(0.0%)	0(0.0%)	0(0.0%)	0(0.0%)	0(0.0%)	0(0.0%)
FBXW7	0(0.0%)	0(0.0%)	0(0.0%)	0(0.0%)	0(0.0%)	0(0.0%)
FGFR1	0(0.0%)	0(0.0%)	0(0.0%)	0(0.0%)	0(0.0%)	0(0.0%)
FGFR2	0(0.0%)	0(0.0%)	0(0.0%)	0(0.0%)	0(0.0%)	0(0.0%)
FGFR3	0(0.0%)	0(0.0%)	0(0.0%)	0(0.0%)	0(0.0%)	0(0.0%)
FLT3	0(0.0%)	0(0.0%)	0(0.0%)	0(0.0%)	0(0.0%)	0(0.0%)
GNAS	0(0.0%)	0(0.0%)	0(0.0%)	0(0.0%)	0(0.0%)	0(0.0%)
HNF1A	0(0.0%)	0(0.0%)	0(0.0%)	0(0.0%)	0(0.0%)	0(0.0%)
HRAS	0(0.0%)	0(0.0%)	0(0.0%)	0(0.0%)	0(0.0%)	0(0.0%)
IDH1	0(0.0%)	0(0.0%)	0(0.0%)	0(0.0%)	0(0.0%)	0(0.0%)
JAK3	0(0.0%)	0(0.0%)	0(0.0%)	0(0.0%)	0(0.0%)	0(0.0%)
KDR	0(0.0%)	0(0.0%)	0(0.0%)	0(0.0%)	0(0.0%)	0(0.0%)
KIT	0(0.0%)	0(0.0%)	0(0.0%)	0(0.0%)	0(0.0%)	0(0.0%)
KRAS	4(5.3%)	1(6.3%)	0(0.0%)	1(9.1%)	2(5.1%)	0(0.0%)
MET	0(0.0%)	0(0.0%)	0(0.0%)	0(0.0%)	0(0.0%)	0(0.0%)
MLH1	0(0.0%)	0(0.0%)	0(0.0%)	0(0.0%)	0(0.0%)	0(0.0%)
MPL	0(0.0%)	0(0.0%)	0(0.0%)	0(0.0%)	0(0.0%)	0(0.0%)
NOTCH1	0(0.0%)	0(0.0%)	0(0.0%)	0(0.0%)	0(0.0%)	0(0.0%)
NPM1	0(0.0%)	0(0.0%)	0(0.0%)	0(0.0%)	0(0.0%)	0(0.0%)
NRAS	0(0.0%)	0(0.0%)	0(0.0%)	0(0.0%)	0(0.0%)	0(0.0%)
PDGFRA	0(0.0%)	0(0.0%)	0(0.0%)	0(0.0%)	0(0.0%)	0(0.0%)
PIK3CA	2(2.6%)	0(0.0%)	0(0.0%)	0(0.0%)	2(5.1%)	0(0.0%)
PTEN	1(1.3%)	0(0.0%)	0(0.0%)	0(0.0%)	1(2.6%)	0(0.0%)
PTPN11	0(0.0%)	0(0.0%)	0(0.0%)	0(0.0%)	0(0.0%)	0(0.0%)
RB1	0(0.0%)	0(0.0%)	0(0.0%)	0(0.0%)	0(0.0%)	0(0.0%)
RET	0(0.0%)	0(0.0%)	0(0.0%)	0(0.0%)	0(0.0%)	0(0.0%)
SMAD4	1(1.3%)	0(0.0%)	0(0.0%)	0(0.0%)	1(2.6%)	0(0.0%)
SMARCB1	0(0.0%)	0(0.0%)	0(0.0%)	0(0.0%)	0(0.0%)	0(0.0%)
SMO	0(0.0%)	0(0.0%)	0(0.0%)	0(0.0%)	0(0.0%)	0(0.0%)
SRC	0(0.0%)	0(0.0%)	0(0.0%)	0(0.0%)	0(0.0%)	0(0.0%)
STK11	0(0.0%)	0(0.0%)	0(0.0%)	0(0.0%)	0(0.0%)	0(0.0%)
TP53	17(22.4%)	2(12.5%)	2(25.0%)	6(54.5%)	5(12.8%)	2(100.0%)
VHL	0(0.0%)	0(0.0%)	0(0.0%)	0(0.0%)	0(0.0%)	0(0.0%)

**Table 6:** Mutations (including Missense point mutations/deletion/insertion) frequencies in 45 genes (737 loci) of human lung cancer samples from heavy smokers (smoking index> = 400), light smokers (smoking index<400) and non-smokers.

Genes	Number of samples with mutations in 76 samples (Mutation frequency)	Number of samples of heavy smokers with mutations (Mutation frequency in 17 samples)	Number of samples of light smokers samples with mutations (Mutation frequency in 6 samples)	Number of samples of non-smoker samples with mutations (Mutation frequency in 52 samples)	Number of unknown samples with mutations (Mutation frequency in 1 sample)
ABL1	0(0.0%)	0(0.0%)	0(0.0%)	0(0.0%)	0(0.0%)
AKT1	0(0.0%)	0(0.0%)	0(0.0%)	0(0.0%)	0(0.0%)
ALK	0(0.0%)	0(0.0%)	0(0.0%)	0(0.0%)	0(0.0%)
APC	0(0.0%)	0(0.0%)	0(0.0%)	0(0.0%)	0(0.0%)
ATM	0(0.0%)	0(0.0%)	0(0.0%)	0(0.0%)	0(0.0%)
BRAF	2(2.6%)	1(5.9%)	0(0.0%)	1(1.9%)	0(0.0%)
CDH1	0(0.0%)	0(0.0%)	0(0.0%)	0(0.0%)	0(0.0%)
CDKN2A	0(0.0%)	0(0.0%)	0(0.0%)	0(0.0%)	0(0.0%)
CSF1R	0(0.0%)	0(0.0%)	0(0.0%)	0(0.0%)	0(0.0%)
CTNNB1	3(3.9%)	0(0.0%)	0(0.0%)	3(5.8%)	0(0.0%)
EGFR	32(42.1%)	3(17.6%)	2(33.3%)	27(51.9%)	0(0.0%)
ERBB2	1(1.3%)	0(0.0%)	0(0.0%)	1(1.9%)	0(0.0%)
ERBB4	0(0.0%)	0(0.0%)	0(0.0%)	0(0.0%)	0(0.0%)
FBXW7	0(0.0%)	0(0.0%)	0(0.0%)	0(0.0%)	0(0.0%)
FGFR1	0(0.0%)	0(0.0%)	0(0.0%)	0(0.0%)	0(0.0%)
FGFR2	0(0.0%)	0(0.0%)	0(0.0%)	0(0.0%)	0(0.0%)
FGFR3	0(0.0%)	0(0.0%)	0(0.0%)	0(0.0%)	0(0.0%)
FLT3	0(0.0%)	0(0.0%)	0(0.0%)	0(0.0%)	0(0.0%)
GNAS	0(0.0%)	0(0.0%)	0(0.0%)	0(0.0%)	0(0.0%)
HNF1A	0(0.0%)	0(0.0%)	0(0.0%)	0(0.0%)	0(0.0%)
HRAS	0(0.0%)	0(0.0%)	0(0.0%)	0(0.0%)	0(0.0%)
IDH1	0(0.0%)	0(0.0%)	0(0.0%)	0(0.0%)	0(0.0%)
JAK3	0(0.0%)	0(0.0%)	0(0.0%)	0(0.0%)	0(0.0%)
KDR	0(0.0%)	0(0.0%)	0(0.0%)	0(0.0%)	0(0.0%)
KIT	0(0.0%)	0(0.0%)	0(0.0%)	0(0.0%)	0(0.0%)
KRAS	4(5.3%)	3(17.6%)	0(0.0%)	1(1.9%)	0(0.0%)
MET	0(0.0%)	0(0.0%)	0(0.0%)	0(0.0%)	0(0.0%)
MLH1	0(0.0%)	0(0.0%)	0(0.0%)	0(0.0%)	0(0.0%)
MPL	0(0.0%)	0(0.0%)	0(0.0%)	0(0.0%)	0(0.0%)
NOTCH1	0(0.0%)	0(0.0%)	0(0.0%)	0(0.0%)	0(0.0%)
NPM1	0(0.0%)	0(0.0%)	0(0.0%)	0(0.0%)	0(0.0%)
NRAS	0(0.0%)	0(0.0%)	0(0.0%)	0(0.0%)	0(0.0%)
PDGFRA	0(0.0%)	0(0.0%)	0(0.0%)	0(0.0%)	0(0.0%)
PIK3CA	2(2.6%)	1(5.9%)	0(0.0%)	1(1.9%)	0(0.0%)
PTEN	1(1.3%)	0(0.0%)	0(0.0%)	1(1.9%)	0(0.0%)
PTPN11	0(0.0%)	0(0.0%)	0(0.0%)	0(0.0%)	0(0.0%)
RB1	0(0.0%)	0(0.0%)	0(0.0%)	0(0.0%)	0(0.0%)
RET	0(0.0%)	0(0.0%)	0(0.0%)	0(0.0%)	0(0.0%)
SMAD4	1(1.3%)	0(0.0%)	0(0.0%)	1(1.9%)	0(0.0%)
SMARCB1	0(0.0%)	0(0.0%)	0(0.0%)	0(0.0%)	0(0.0%)
SMO	0(0.0%)	0(0.0%)	0(0.0%)	0(0.0%)	0(0.0%)
SRC	0(0.0%)	0(0.0%)	0(0.0%)	0(0.0%)	0(0.0%)
STK11	0(0.0%)	0(0.0%)	0(0.0%)	0(0.0%)	0(0.0%)
TP53	17(22.4%)	7(41.2%)	1(16.7%)	9(17.3%)	0(0.0%)
VHL	0(0.0%)	0(0.0%)	0(0.0%)	0(0.0%)	0(0.0%)

### Missense mutation distribution in the exons and functional domains of EGFR

**Figure 2:**
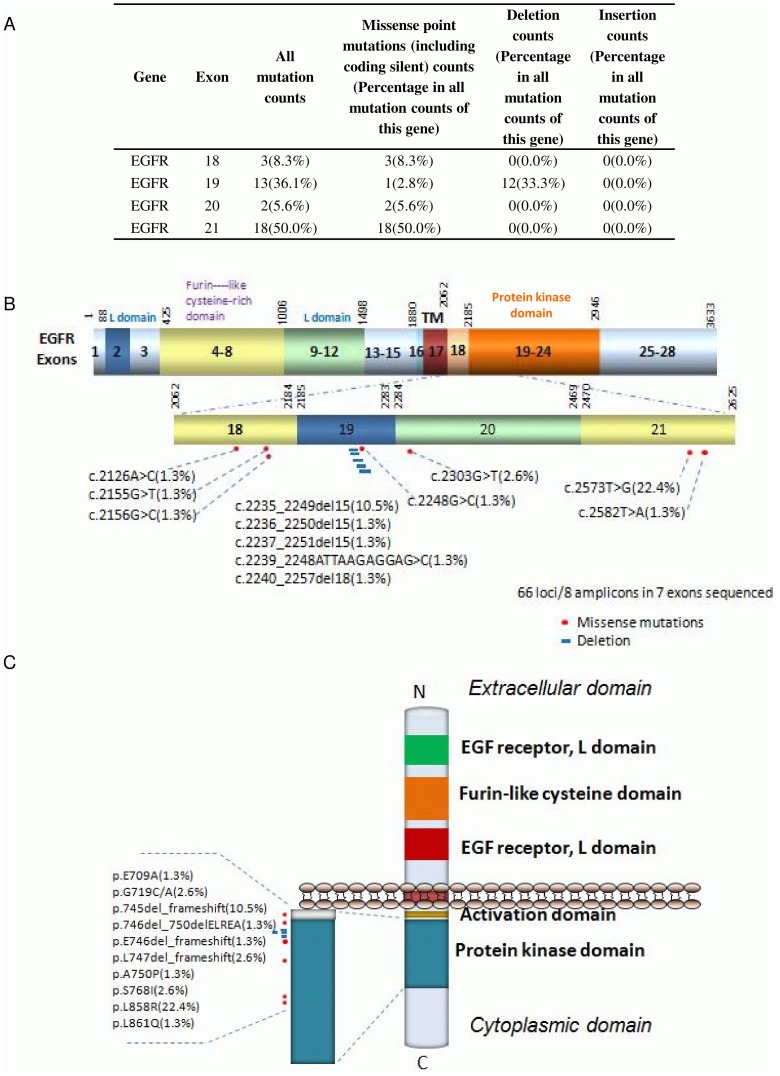
Missense mutation distribution in the exons and function domains of EGFR. A. Frequencies of detected mutations in different exons. B. Mutation distribution in exons. C. Mutation distribution in functional domains.

Out of 76 sequenced lung cancer samples, 36.1% of EGFR mutations were missense along exon 19, 50.0% were missense along exon 21, 5.6% along exon 20 and 8.3% along exon 18 (**Fig. 2A**). These mutations were in and around the tyrosine kinase domain of EGFR (**Fig. 2B** **–** **3C**). Activating mutations in the tyrosine kinase domain of the *EGFR* gene stimulates protein tyrosine kinase, which leads to activation of signaling pathways associated with cell growth and survival. Mutations in the extracellular domain of EGFR is often associated with the amplification of genes in other cancers [12]. 57.7% of EGFR-associated lung cancers were adenocarcinomas (**Table 2**) and 86.7% of EGFR mutations associated with ‘high differentiation’ cancers (**Table 3**). In our sample set 50% of EGFR-associated lung cancers metastasized to local regions, 27.3% to lymphs and 46.2% of cancers metastasized to distant organs in our sample set (**Table 5**).

**Figure 3:**
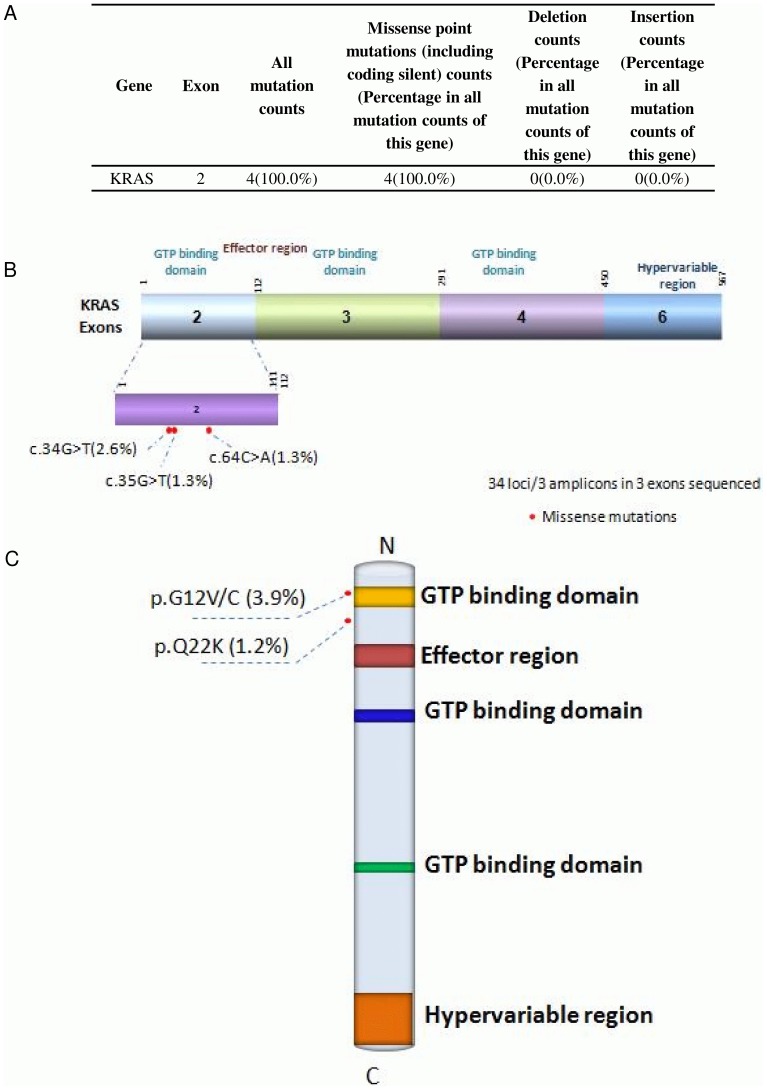
Missense mutation distribution in the exons and function domains of KRAS. A. Frequencies of detected mutations in different exons. B. Mutation distribution in exons. C. Mutation distribution in functional domains.

### Missense mutation distribution in the exons and functional domains of KRAS

Out of 76 sequenced Lung cancer samples, 100% of KRAS mutations were missense along exon 2 (**Fig. 3A**). The 34G>T mutations result in an amino acid substitution at position 12 in KRAS, from a glycine (G) to a cysteine (C) or a valine (V). The 64C>A mutation results in an amino acid substitution at position 22 from a glutamine (Q) to a lysine (K) in KRAS. All of these amino acid substitutions occurred along the GTP binding domain of KRAS (**Fig. 3A–C**). KRAS binds to GTP in the active state and possesses an intrinsic enzymatic activity which cleaves the terminal phosphate of the nucleotide, converting it to GDP. Upon conversion of GTP to GDP, KRAS is turned off [13]. The result of these mutations is constitutive activation of ***KRAS*** signaling pathways. Once it is turned on, it recruits and activates proteins necessary for the propagation of growth factor and other receptors' signal such as c-Raf and PI3-kinase [13]. 7.7% of KRAS-associated lung cancers were adenocarcinomas (**Table 2**) and 6.1% of KRAS mutations associated with ‘low differentiation’ cancers and 7.4% of KRAS mutations were ‘mid differentiation’ cancers (**Table 3**). In our sample set 6.3% of KRAS-associated lung cancers metastasized to local regions, 9.1% to lymphs and 5.1% of cancers metastasized to distant organs in our sample set (**Table 5**).

### Missense mutation distribution in the exons and functional domains of TP53

**Figure 4:**
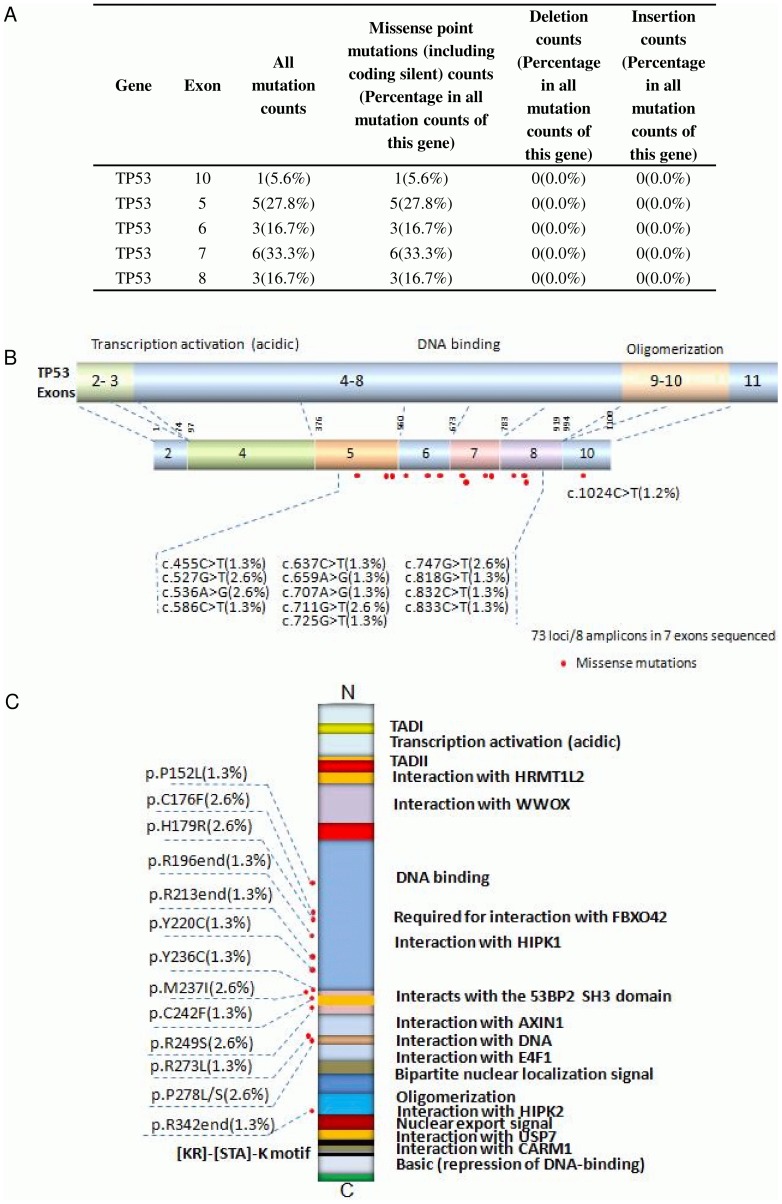
Missense mutation distribution in the exons and function domains of TP53. A. Frequencies of detected mutations in different exons. B. Mutation distribution in exons. C. Mutation distribution in functional domains.

Abnormality of the TP53 gene is one of the most significant events in lung cancers and plays an important role in the tumorigenesis of lung epithelial cells. The p53 tumor suppressor gene is located on 17p13 chromosome and spans 20 kb genomic DNA encompassing 11 exons that encodes for a 53KD phosphoprotein [14]. Most TP53 mutations cluster in the TP53 DNA-binding domain, which encompasses exons 5 through 8 and spans approximately 180 codons or 540 nucleotides and is not limited to a few particular sequences or codons along this gene [15]. TP53 incurred several deleterious mutations in our sample set of 76 lung cancers, mostly along the DNA-binding domain encoded from exon 5 (27.8%), 6 (16.7%), 7 (33.3%), 8 (16.7%), and along the oligomerization domain encoded from exon 10 (15.6%) (**Fig. 4A–C**). Most TP53 missense mutations lead to the synthesis of a stable protein, which lacks its specific DNA-binding and transactivation function and accumulates in the nucleus of cells. Such mutant proteins become inactive and lack the ability to transactivate the downstream target genes that regulate cell cycle and apoptosis [16]. Apart from these mutations affecting the role of TP53 as a tumor-suppressor protein, TP53 mutations also endow the mutant protein with ‘gain-of-function’ (GOF) activities, which can contribute actively to various stages of tumor progression, including distant metastases, and to increased resistance to anticancer treatments [17]–[19]. 50.0% of TP53-associated lung cancers were squamous cell carcinoma (**Table 2**) and 20.0% of TP53 mutations associated with ‘high differentiation’ cancers and 25.9% of TP53 mutations were ‘mid differentiation’ cancers (**Table 3**). In our sample set 25.0% of TP53-associated lung cancers metastasized to local regions, 54.5% to lymphs and 12.8% of cancers metastasized to distant organs in our sample set (**Table 5**).

### Multiple mutations and mutation hot spots in human lung cancers

Clinical success with individualized combination therapy relies on the identification of mutational combinations and patterns for co-administration of a single or combination of target agents against the detected mutational combinations. Some of the mutations detected in our tumor group through sequencing analysis were not only recurrent and frequent but also occurred in combination with other mutations. Lung cancers in our sample set contained the following: 64.5% of samples had at least one or more missense mutations, 19.7% had at least two or more missense mutations, 3.9% had at least three or more missense mutations, 1.3% had at least four or more missense mutations, and 35.5% of samples incurred no deleterious mutations in any of the screened 13,500 loci of the potential tumor suppressor and oncogenes (**Table 7**).

**Table 7:** Single and multiple missense mutations (including coding silent/deletion/insertion) in genes of 76 human lung cancer samples.

Mutations combination (including Missense point mutations/deletion/insertion)	Number of samples with mutation combination	Percentage in all sequenced samples
Single and more	49	64.50%
Double and more	15	19.70%
Three and more	3	3.90%
Four and more	1	1.30%
Five and more	0	0.00%
No missense, deletion, insert or substitution-nonsense	27	35.50%

## Discussion

As lung cancer is the most prevalent cancer and leading cause of cancer deaths worldwide, ongoing efforts are aimed to improve prevention, diagnosis, and effective treatment options for patients with lung cancer. Currently there are a range of treatment options for lung cancer patients, with surgery being the most effective for treatment of NSCLCs, and chemotherapy with or without radiation therapies as the standard treatment for SCLCs. Because most SCLCs metastasize early to distant organs, surgery is often ineffective in curing this cancer. NSCLCs, on the other hand, are more likely to remain localized during development, and are thus are more effectively treated with surgical intervention. Additionally, SCLCs are typically much more sensitive to chemotherapy and/or radiation therapy than are NSCLCs [20], [21]. One challenge in proper classification and treatment of lung cancer is the extreme heterogeneity caused by differing genetic, biological, and clinical properties, including response to treatment, with over 50 histological variants recognized by the WHO typing system [22], [23]. Because of this, correct classification of lung cancer cases is necessary to assure that patients receive optimum management [24].

Due to of these various levels of heterogeneity, generalized treatments may be less effective. Alternatively targeted therapy, which involves the usage of specially designed drugs to selectively target molecular pathways correlated with the malignant phenotype of lung cancer cells, may be more useful [25]. Several genes commonly found to be mutated in various lung cancers have been reported, including ALK/ELM4 fusion, K-*ras*, EGFR, VEGF, and p53, yet the entire genetic profile of each form is still not been fully defined [3]. This indicates the necessity of sequencing individual human lung cancers in order to match the use of a single targeted drug or two or more targeted drugs in combination against individual lung cancer-specific mutations. In this study we have used Ion Ampliseq Cancer Panel to sequence 13,500 loci in 45 cancer-related genes, mainly oncogenes and tumor suppressor genes, of 76 human lung cancer samples. We identified frequent mutations in a group of genes, including EGFR, KRAS, and TP53 (**Table 2**). Although most of these genes were already known to be associated with lung cancers, the mutated points and the associated mutations in other genes were different in our sample set (**Tables 7**).

As there is increasing awareness about the changes in lung cancer cells in recent times, newer drugs that specifically target these changes have been developed. These targeted drugs either work synergistically with the chemotherapy drugs or by themselves with much lesser toxicity due to a selective effect as an alternative to a more systemic modulation of proteins associated with oncogenesis. EGFR inhibitors (Afatinib, Erlotinib, and Gefitinib) and VEGF inhibitors (Bevacizumab) are currently used for target therapies for NSCLC patients with mutations in the VEGF and EGFR [26]. Erlotinib is a drug that blocks EGFR from signaling the cell to grow. It prevents the progression of lung cancer, specifically in non-smoking women, and is mostly used in advanced NSCLC treatment that was not responsive to chemotherapy. It is also used as the first treatment in patients whose cancers have a mutation in the *EGFR* gene [27]. Cetuximab is a monoclonal antibody that targets EGFR which is also used in advanced NSCLC in combination with standard chemotherapy as part of first-line treatment [28]. Like erlotinib, afatinib is a drug that blocks the growth signal from EGFR and used for advanced NSCLCs that have mutations in the *EGFR* gene [29]. Some younger, non-smokers with adenocarcinomas are found to have an ALK/EML4 fusion oncogene which is currently a target for the drug Crizotinib [30]. Other drugs currently used to treat lung cancers are not gene-specific, and instead target general molecular pathways like folate anitmetabolites (methotrexate and pemetrexed), mitotic inhibitors (docetaxel, piclitaxel, and vinorelbine), topoisomerase inhibitors (etopophos and topotecan), and nucleoside analogs which interfere with DNA synthesis (carboplatin, cisplatin, and gemciabine) [31], [32]. Inhibitors of EGFR-directed tyrosine kinase are established to be an effective treatment option for advanced NSCLC not responding to chemotherapy. However, EGFR-directed monoclonal antibodies in combination with platinum-based first-line chemotherapy, cetuximab combined with cisplatin/vinorelbine and bevacizumab in combination with platinum-based chemotherapy resulted in better survival compared to chemotherapy alone in patients with advanced EGFR-positive NSCLC [33]. Other targeted therapies including dual and multi-kinase inhibitors are in earlier stages of clinical development [34].

With the accumulation of knowledge and experience in next generation technologies, it is necessary to expand our understanding in the sensitivity of specific mutations to individualized therapies. Therefore, gathering a complete profile of mutations in lung cancers for the application of personalized and tailored targeted therapy is critical to develop future cancer treatments. We believe a faster and cost effective genotyping tool such as Ion Torrent sequencing technology will be greatly beneficial for the assignment of such specific therapeutics in the near future use for lung cancers.

## Materials and Methods

### Ethics statement

The study has been approved by the Human Research Ethics Committee of the First Affiliated Hospital of Dalian Medical University, China. For Formalin fixed and paraffin embedded (FFPE) tumor samples from the tumor tissue bank at the Department of Pathology of the hospital, the institutional ethics committee waived the need for consent. All samples and medical data used in this study have been irreversibly anonymized.

### Patient information

Tumor samples used in the study were collected from the First Affiliated Hospital of Dalian Medical University, China. A total of 76 FFPE tumor samples from lung cancer patients were analyzed. The mean age of the 76 patients was 61 years (range: 28–80 years). Of these, 40 patients were male with a mean age of 61 years (range: 28–80 years), and 36 patients were female with a mean age of 61 (range: 36–75 years). Tumor samples used in the study were collected from the First Affiliated Hospital of Dalian Medical University, China. A total of 76 FFPE tumor samples from lung cancer patients were analyzed. The mean age of the 76 patients was 61 years (range: 28–80 years). Of these, 40 patients were male with a mean age of 61 years (range: 28–80 years), and 36 patients were female with a mean age of 61 (range: 36–75 years). 33 of the 76 patients (20 men, 13 women) were graded as low pathologic differentiation; 27 (14 men, 13 women) at mid, 15 (6 men, 9 women) at high, and 1 women of unknown differentiation. AJCC cancer staging is as follows: 0 patients at I or Ia; 18 (10 men, 8 women) at stage Ib; 3 (2 men, 1 woman) at stage IIa; 9 (5 men, 4 women) at stage IIb; 26 (14 men, 12 women) at stage IIIa; 8 (4 men, 4 women) at stage IIIb; 0 patients at stage IIIc; and 12 (5 men, 7 women) at stage IV. Out of the total 76 patients, 16 of the 40 men reported no history of smoking, whereas none of the 36 women reported to be smokers; 6 men reported light smoking; 17 men reported heavy smoking, and 1 man with an unknown smoking history.

### DNA preparation

DNA was isolated from FFPE samples after deparaffinization and extraction of 3–5 µm thick paraffin sections in xylene, using the QIAamp DNA Mini Kit (Qiagen) per the manufacturer's instructions.

### Ion torrent PGM library preparation and sequencing

An Ion Torrent adapter-ligated library was made following the manufacturer's protocol for the Ion AmpliSeq Library Kit 2.0 (Life Technologies) (Part #4475345 Rev. A). Briefly, 50 ng pooled amplicons were end-repaired, and DNA ligase was used to ligate Ion Torrent adapters P1 and A. After purification with AMPure beads (Beckman Coulter, Brea, CA, USA), adapter-ligated products were nick-translated and PCR-amplified for a total of 5 cycles. AMPure beads (Beckman Coulter) were used to purify the resulting library, and an Agilent 2100 BioAnalyzer (Agilent Technologies) and Agilent BioAnalyzer DNA High-Sensitivity LabChip (Agilent Technologies) were used to determine the concentration and size of the library.

Sample emulsion PCR, emulsion breaking, and enrichment were performed using the Ion PGM 200 Xpress Template Kit (Part #4474280 Rev. B), according to the manufacturer's instructions. Briefly, an input concentration of one DNA template copy/Ion Sphere Particles (ISPs) was added to the emulsion PCR master mix and the emulsion generated using an IKADT-20 mixer (Life Technologies). Next, ISPs were recovered and template-positive ISPs were enriched for use with Dynabeads MyOne Streptavidin C1 beads (Life Technologies). ISP enrichment was confirmed using the Qubit 2.0 fluorometer (Life Technologies). 316 chips were used for sequencing on the Ion Torrent PGM for 65 cycles and the samples were barcoded. Ion PGM 200 Sequencing Kit was used for sequencing reactions, as per the recommended protocol (Part # 4474004 Rev. B). The dataset has been deposited to the NIH Sequence Read Archive, and the accession number is SRP028756 (http://www.ncbi.nlm.nih.gov/Traces/sra/?study=SRP028756).

### Variant calling

Data from the PGM runs were processed initially using the Ion Torrent platform-specific pipeline software Torrent Suite to generate sequence reads, trim adapter sequences, filter, and remove poor signal-profile reads. Initial variant calling from the Ion AmpliSeq sequencing data was generated using Torrent Suite Software v3.0 with a plug-in “variant caller” program. In order to eliminate erroneous base calling, three filtering steps were used to generate final variant calling. The first filter was set at an average total coverage depth >100, each variant coverage >20, a variant frequency of each sample >5, and P-value<0.01. In order to eliminate error base calling, several filtering steps were used to generate final variant calling (**Fig. S1.**). The first filter was set at an average depth of total coverage of >100, an each variant coverage of >20, a variant frequency of each sample >5 and P-value <0.01. The second filter was employed by visually examining mutations using Integrative Genomics Viewer (IGV) software (http//www.broadinstitute.org/igv) or Samtools software SAMtools software (http://samtools.sourceforge.net), as well as by filtering out possible strand-specific errors, ie. a mutation was only detected in either “+” or “-” strand, but not in both strands of DNA. The third filtering step was set as variants within 727 hotspots, according to the manufacturer' instructions. The last filter step was eliminate variants in amplicon AMPL339432 (PIK3CA, exon13, chr3:178938822-178938906), which is not unique matched in human genome. From our sequencing runs using the Ion Ampliseq Cancer Panel, false deletion data were generated from the JAK2 gene locus and thus the sequencing data from this locus were excluded from further analysis.

### Somatic mutations

Detected mutations were compared to variants in the 1000 Genomes Project [35] and 6500 exomes of the National Heart, Lung, and Blood Institute Exome Sequencing Project [36] to distinguish somatic mutations and germline mutations.

### Bioinformatical and experimental validation applied

We used the COSMIC[37] (version 64), MyCancerGenome database (http://www.mycancergenome.org/) and some publications to assess reappearance mutations in lung cancer (**Table S1**). Additionally, some detected missense mutations were confirmed by Sanger's sequencing (**Fig. S2**).

### Statistical analysis

We select reappearance somatic missense/in-del mutations of lung cancer to do the statistical analysis.

## Supporting Information

Figure S1 Filter process of variants. Note: (a) Strand-biased variants were eliminated using Integrative Genomics Viewer (IGV) software (http//www.broadinstitute.org/igv); (b) Variants in AMPL339432 should be eliminated, because this amplicon is not unique matched to PIK3CA in human genome; (c) All of our statistical analysis was based on the data in blue box.
(DOCX)

Figure S2 Sanger validations of 15 variants.
(DOC)

Table S1 Frequencies of point mutations, insertion and deletion mutations in 737 loci of 76 human lung cancers.
(DOCX)
