# Supplementary figures and images for: Frequent Mutations in EGFR, KRAS and TP53 Genes in Human Lung Cancer Tumors Detected by Ion Torrent DNA Sequencing
Source: PLoS One. 2014 Apr 23;9(4):e95228. doi: 10.1371/journal.pone.0095228 (PMC3997391; doi:10.1371/journal.pone.0095228)

**Figure S1.**


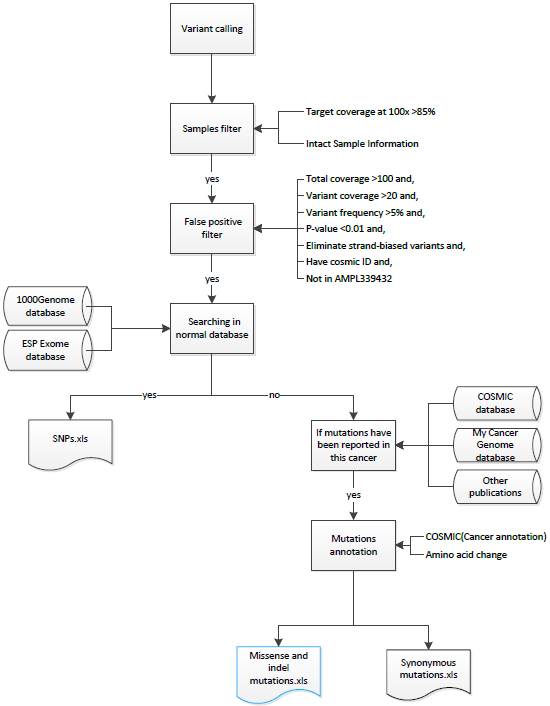

Supplement: Figure S1 — Filter process of variants. Note: (a) Strand-biased variants were eliminated using Integrative Genomics Viewer (IGV) software (http//www.broadinstitute.org/igv); (b) Variants in AMPL339432 should be eliminated, because this amplicon is not unique matched to PIK3CA in human genome; (c) All of our statistical analysis was based on the data in blue box. (DOCX) [file pone.0095228.s001.docx]

**Figure S2. Sanger sequencing**

1.


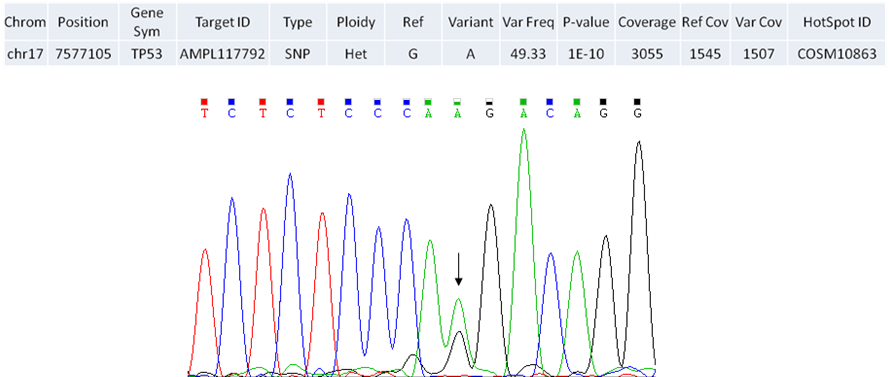


2.


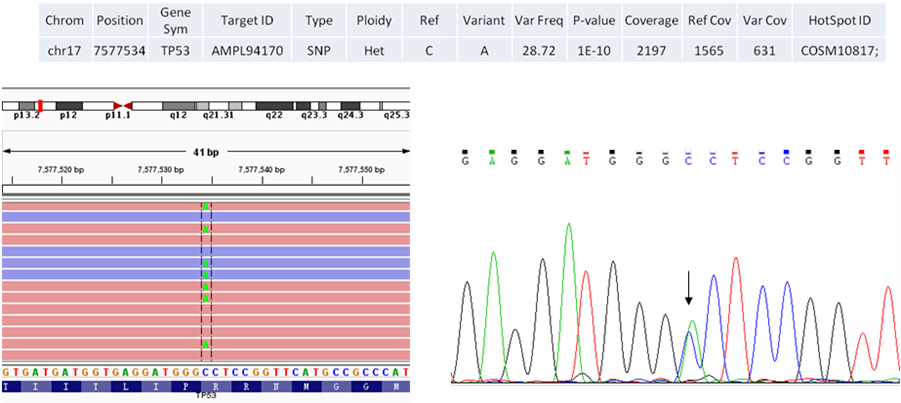


3.


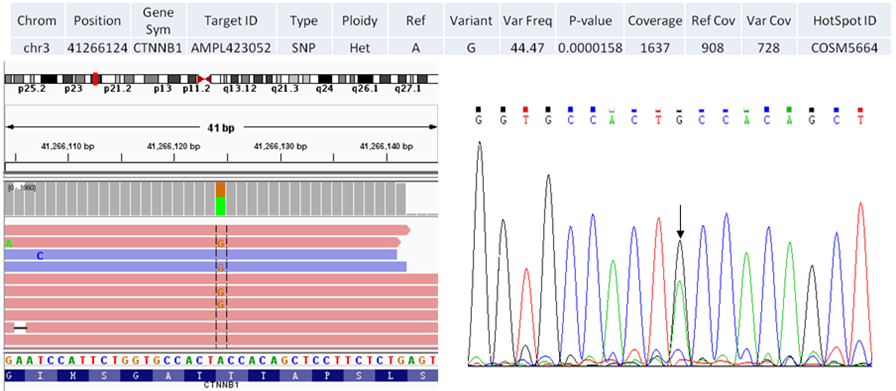


4.


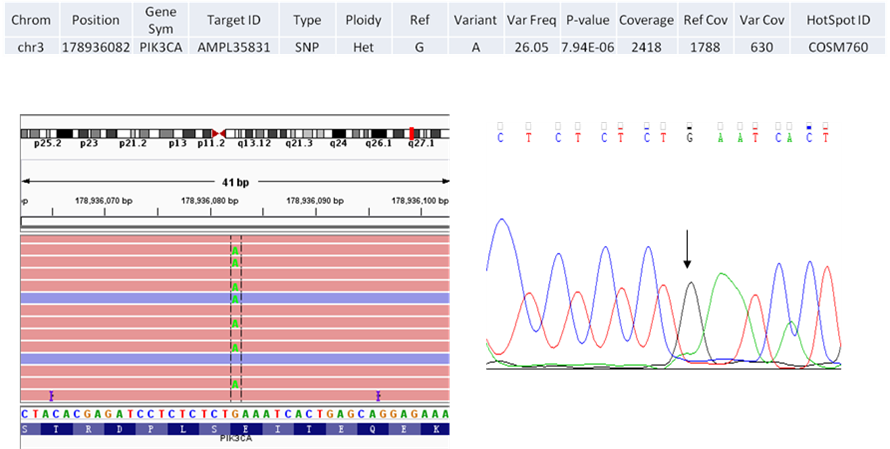


5.


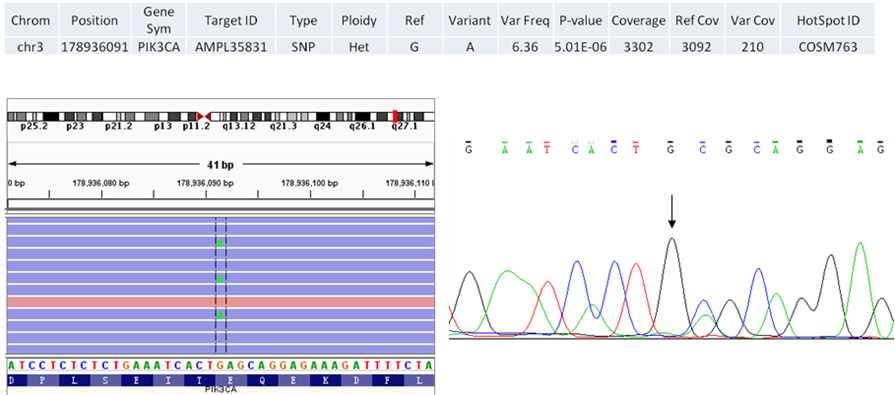


6.


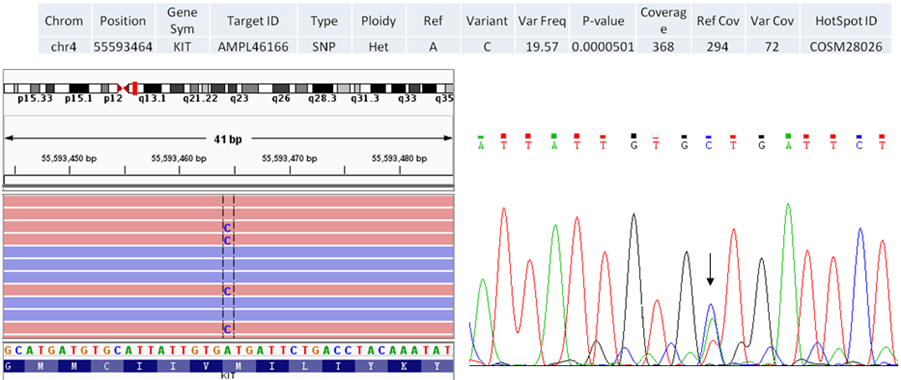


7.


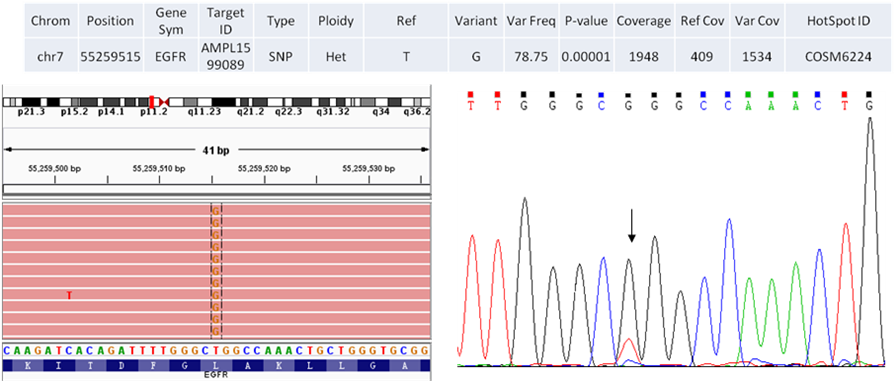


8.
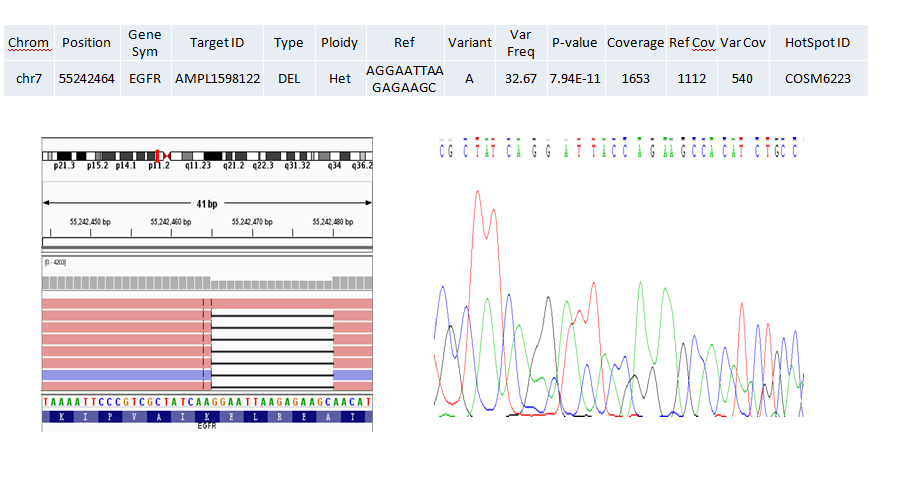


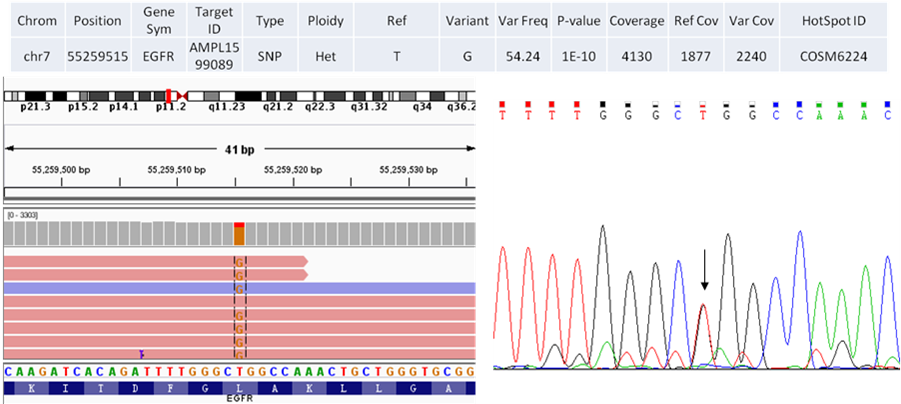
9.

10.


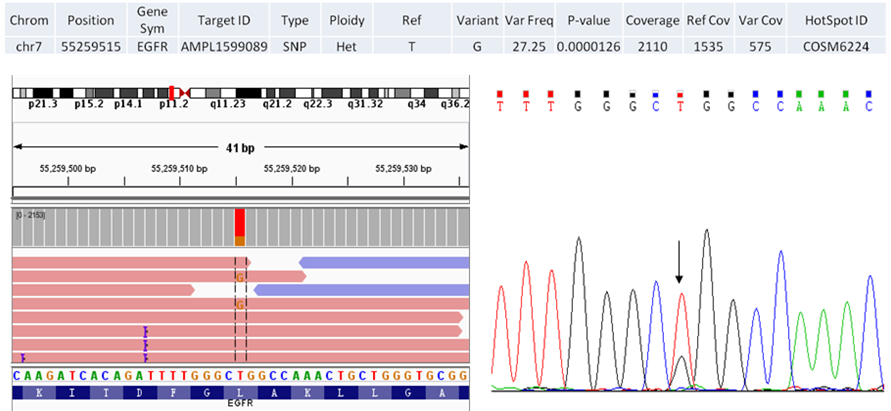


11.
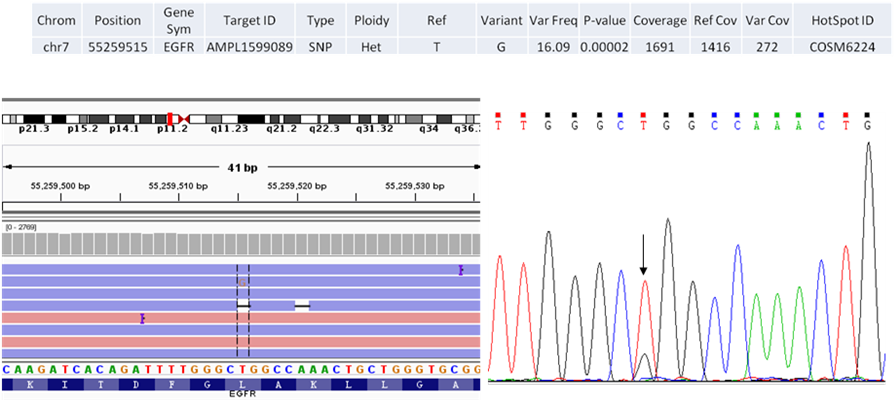


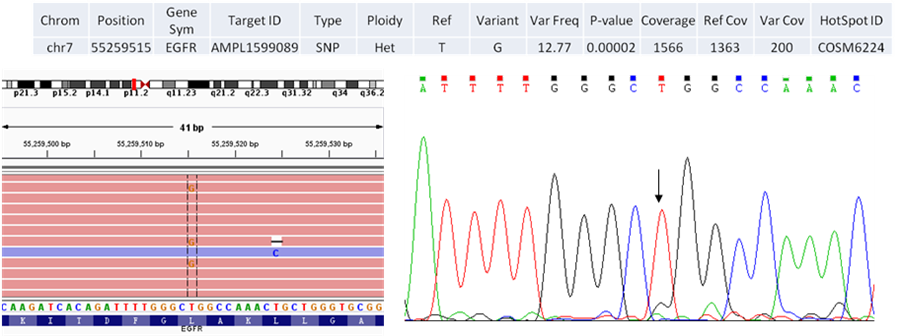
 #12

13.
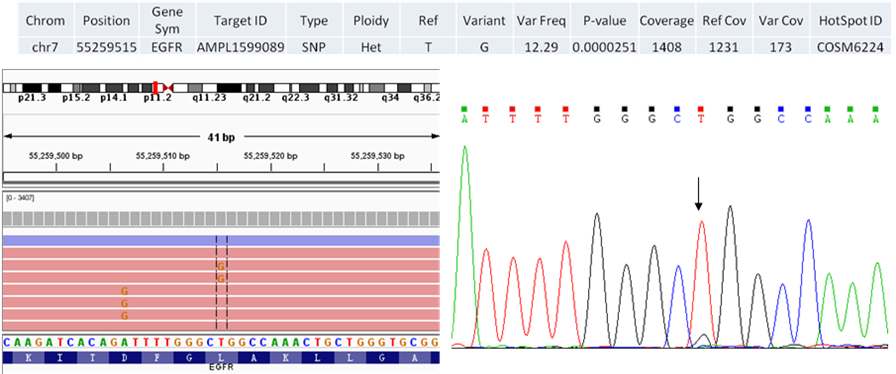


14.
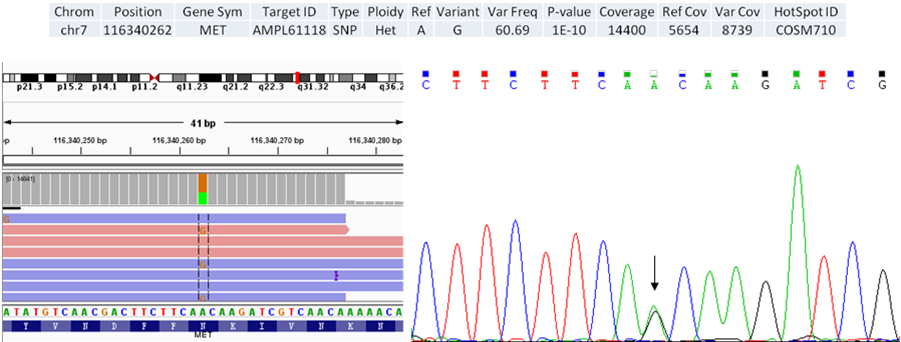


15.
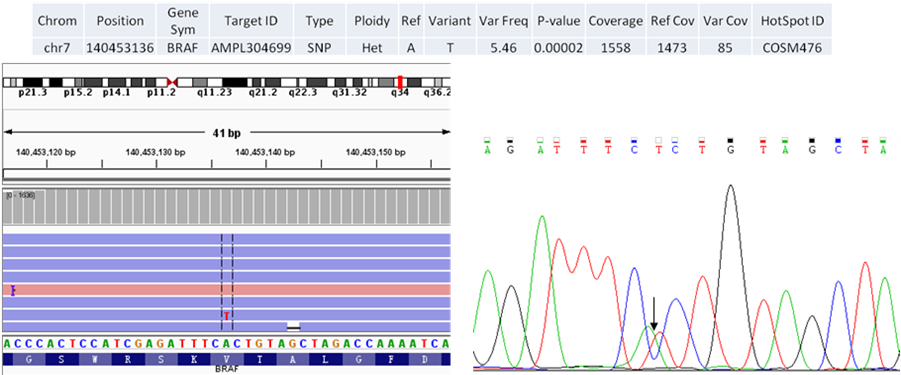

Supplement: Figure S2 — Sanger validations of 15 variants. (DOC) [file pone.0095228.s002.doc]
