# Supplementary material for: Frequent Mutations in EGFR, KRAS and TP53 Genes in Human Lung Cancer Tumors Detected by Ion Torrent DNA Sequencing
Source: PLoS One. 2014 Apr 23;9(4):e95228. doi: 10.1371/journal.pone.0095228 (PMC3997391; doi:10.1371/journal.pone.0095228)
Supplement: Table S1 — Frequencies of point mutations, insertion and deletion mutations in 737 loci of 76 human lung cancers. (DOCX) [file pone.0095228.s003.docx]

**Table S1. Frequencies of point mutations, insertion and delection mutations in 737 loci of 76 human lung cancer.**

| **Gene Mutations** | **Number of samples with this mutation site** | **Number of samples with this mutation gene** | **Mutation Frequency** | **Gene mutation frequency in publications** | **Site mutation frequency in gene in publications** |
| --- | --- | --- | --- | --- | --- |
| BRAF c.1799T>A | 2 | 2 | 2.6% | 1–4%（mycancergenome） | 55.00%（mycancergenome） |
| CTNNB1 c.110C>T | 1 | 3 | 3.9% | 2-4%^[^[^4^](#_ENREF_1)^]^ | - |
| CTNNB1 c.121A>G | 1 |  |  |  | - |
| CTNNB1 c.133T>C | 1 |  |  |  | - |
| EGFR c.2126A>C | 1 | 32 | 42.1% | ~35%（mycancergenome） | 3.00%（mycancergenome） |
| EGFR c.2155G>T | 1 |  |  |  | - |
| EGFR c.2156G>C | 1 |  |  |  | - |
| EGFR c.2235_2249del15 | 8 |  |  |  | exon 19 deletion: 48.00%（mycancergenome） |
| EGFR c.2236_2250del15 | 1 |  |  |  |  |
| EGFR c.2237_2251del15 | 1 |  |  |  |  |
| EGFR c.2240_2257del18 | 1 |  |  |  |  |
| EGFR c.2248G>C | 1 |  |  |  | - |
| EGFR c.2303G>T | 2 |  |  |  | - |
| EGFR c.2573T>G | 17 |  |  |  | 43.00%（mycancergenome） |
| EGFR c.2582T>A | 1 |  |  |  | 2.00%（mycancergenome） |
| EGFR c.2239_2248ATTAAGAGGAG>C | 1 |  |  |  | - |
| ERBB2 c.2326_2327insTGT | 1 | 1 | 1.3% | 2–4% | exon 20 insertion: 83–100%（mycancergenome） |
| KRAS c.34G>T | 2 | 4 | 5.3% | 15–25%（mycancergenome） | 42.00%（mycancergenome） |
| KRAS c.35G>T | 1 |  |  |  | 20.00%（mycancergenome） |
| KRAS c.64C>A | 1 |  |  |  | ～3.5% Collaborative mutations^[^[^5^](#_ENREF_2)^]^ |
| PIK3CA c.1624G>A | 1 | 2 | 2.6% | 1–3%（mycancergenome） | 8.9%（mycancergenome） |
| PIK3CA c.3140A>G | 1 |  |  |  | 12.90%（mycancergenome） |
| PTEN c.202T>C | 1 | 1 | 1.3 % | 4–8%（mycancergenome） | - |
| SMAD4 c.1082G>A | 1 | 1 | 1.3% | - | - |
| TP53 c.1024C>T | 1 | 17 | 22.4% | 47%（Japan）^[^[^6^](#_ENREF_3)^]^ | - |
| TP53 c.455C>T | 1 |  |  |  | - |
| TP53 c.527G>T | 2 |  |  |  | - |
| TP53 c.536A>G | 2 |  |  |  | - |
| TP53 c.586C>T | 1 |  |  |  | - |
| TP53 c.637C>T | 1 |  |  |  | - |
| TP53 c.659A>G | 1 |  |  |  | - |
| TP53 c.707A>G | 1 |  |  |  | - |
| TP53 c.711G>T | 2 |  |  |  | - |
| TP53 c.725G>T | 1 |  |  |  | - |
| TP53 c.747G>T | 2 |  |  |  | - |
| TP53 c.818G>T | 1 |  |  |  | - |
| TP53 c.832C>T | 1 |  |  |  | - |
| TP53 c.833C>T | 1 |  |  |  | - |
